# Co-implementing vitamin A supplementation with seasonal malaria chemoprevention in Sokoto State, Nigeria: a feasibility and acceptability study

**DOI:** 10.1186/s12913-022-08264-z

**Published:** 2022-07-05

**Authors:** Olusola Oresanya, Abimbola Phillips, Ekechi Okereke, Abraham Ahmadu, Taiwo Ibinaiye, Madeleine Marasciulo, Charlotte Ward, Olatunde Adesoro, Rilwanu Mohammed, Jamilu Nikau, Chris Osa Isokpunwu, Mohammad Ali Inname, Helen Counihan, Kevin Baker, Kolawole Maxwell, Helen Smith

**Affiliations:** 1 Malaria Consortium Nigeria, Abuja, Nigeria; 2 Malaria Consortium United States, Raleigh, NC 27615 USA; 3 Malaria Consortium United Kingdom, London, UK; 4 National Malaria Elimination Programme, Abuja, Nigeria; 5 Nutrition Division, Federal Ministry of Health, Abuja, Nigeria; 6 Sokoto State Ministry of Health, Sokoto, Nigeria

**Keywords:** Seasonal malaria chemoprevention, Vitamin A supplementation, Integration, Health campaign effectiveness, Micronutrient deficiency, Intervention coverage, Feasibility, Acceptability, SMC plus vitamin A supplementation

## Abstract

**Background:**

Bi-annual high dose vitamin A supplements administered to children aged 6–59 months can significantly reduce child mortality, but vitamin A supplementation (VAS) coverage is low in Nigeria. The World Health Organization recommends that VAS be integrated into other public health programmes which are aimed at improving child survival. Seasonal malaria chemoprevention (SMC) provides a ready platform for VAS integration to improve health outcomes. This study explored the feasibility and acceptability of integrating VAS with SMC in one local government area in Sokoto State.

**Methods:**

A concurrent QUAN-QUAL mixed methods study was used to assess the feasibility and acceptability of co-implementing VAS with SMC in one LGA of Sokoto state. Existing SMC implementation tools and job aids were revised and SMC and VAS were delivered using a door-to-door approach. VAS and SMC coverage were subsequently assessed using questionnaires administered to 188 and 197 households at baseline and endline respectively. The qualitative component involved key informant interviews and focus group discussions with policymakers, programme officials and technical partners to explore feasibility and acceptability. Thematic analysis was carried out on the qualitative data.

**Results:**

At endline, the proportion of children who received at least one dose of VAS in the last six months increased significantly from 2 to 59% (*p* < 0.001). There were no adverse effects on the coverage of SMC delivery with 70% eligible children reached at baseline, increasing to 76% (*p* = 0.412) at endline. There was no significant change (*p* = 0.264) in the quality of SMC, measured by proportion of children receiving their first dose as directly observed treatment (DOT), at baseline (54%) compared to endline (68%). The qualitative findings are presented as two overarching themes relating to feasibility and acceptability of the integrated VAS-SMC strategy, and within each, a series of sub-themes describe study participants’ views of important considerations in implementing the strategy.

**Conclusion:**

This study showed that it is feasible and acceptable to integrate VAS with SMC delivery in areas of high seasonal malaria transmission such as northern Nigeria, where SMC campaigns are implemented. SMC-VAS integrated campaigns can significantly increase vitamin A coverage but more research is required to demonstrate the feasibility of this integration in different settings and on a larger scale.

## Background

Globally, about two billion people are estimated to have micronutrient deficiencies [1] and reports indicate that children from low and middle-income countries are most affected [2]. In Nigeria, vitamin A deficiency is a public health problem and a major risk factor for child survival, increasing the number of fatalities caused by common diseases such as acute gastroenteritis and measles [3, 4]. Children with clinical signs of vitamin A deficiency are at least three times more likely to die than children who are not vitamin A deficient [5].

High dose vitamin A supplementation (VAS) delivered twice per year to children under five years in low and middle-income countries, is a proven low-cost intervention which has been shown to reduce all-cause mortality by about 24 percent [6, 7]. The World Health Organization (WHO) recommends high dose VAS given every 4 to 6 months to children aged 6–59 months who are at risk of vitamin A deficiency [8]. Countries with high under-five mortality rates (more than 70 per 1,000 births) and evidence of vitamin A deficiency among this age group have been identified as priority countries for national VAS programmes [9]. Nigeria, with an under-five mortality rate of 132 per 1000 births [10], is a priority country for national VAS campaigns. These are conducted bi-annually within the country, mainly during Maternal, Newborn and Child Health (MNCH) weeks, through a health facility-based approach [11].

According to the 2018 Nigerian National Nutrition and Health Survey (NNHS), only 41 percent of children aged 6 – 59 months received VAS in the six months prior to the survey, with Sokoto and four other States recording less than 15 percent coverage [12]. Between 2014 and 2018, the number of states meeting the minimum threshold of 70 percent coverage at which reductions in child mortality can be expected, reduced from seven to two states in Nigeria. An independent assessment of the MNCH week revealed that none of the states implemented the strategy as per guidelines and no evidence was found that MNCH week significantly contributed to coverage of essential MNCH interventions, including VAS [11]. Addressing the poor VAS coverage among these vulnerable groups is key for child survival in Nigeria and critical for universal health coverage. To promote more equitable access to this life-saving intervention, WHO recommends that VAS be integrated into other public health programmes which are aimed at improving child survival [8], as evidence suggests that integrating interventions for multiple diseases can increase coverage, improve health outcomes and could be cost-effective [13–15].

Seasonal malaria chemoprevention (SMC) provides a ready and viable platform to integrate VAS for higher coverage for children under five years old. SMC involves four-monthly administration of three-day treatment courses of sulfadoxine-pyrimethamine (SP) and amodiaquine (AQ), also called SPAQ, to children 3–59 months. This is where malaria transmission is highly seasonal, usually between July and October, in the Sahel region of sub-Saharan Africa [16]. The goal is to maintain therapeutic antimalarial drug concentrations in the blood throughout the period of greatest risk to prevent malaria [17]. The SMC intervention has been successfully implemented with coverage of more than 80 percent in Sokoto, Zamfara, Katsina, Jigawa, Kebbi and Yobe states with good acceptability [18]. In Nigeria, SMC is delivered during each cycle using a door-to-door approach, where teams of community health workers called community drug distributors (CDDs) visit each household within their assigned catchment areas to administer the first dose of SPAQ as a directly observed treatment (DOT), and provide information to caregivers on how to administer the remaining two subsequent daily doses of AQ.

The SMC platform has been used to increase vaccination rates in children under five by over 50 percent in Mali [19]. A study within northern Nigeria reported that when a lipid-based nutrient supplement (LNS) distribution was integrated with SMC programme, coverage for the LNS was about 85 percent, without any significant effect on SMC coverage, which was also high at 90 percent [20]. SMC uses a door-to-door delivery strategy, giving more children missed by the outreach strategy implemented during the VAS campaigns the opportunity to be reached to receive at least one dose during the year.

Despite the potential benefits of co-implementing VAS with SMC, there are some challenges that need to be addressed. Firstly, the safety of administering VAS via the SMC platform that is designed to target a different age group is a key concern. While SMC targets children aged 3–59 months, VAS is administered to children aged 6–59 months, there is thus the challenge for CDDs to determine which children are eligible for both vitamin A capsules and SPAQ. A second concern is that the interval between treatments with vitamin A capsules and SPAQ varies. VAS should be administered once every four to six months while SMC is given over a three-day period monthly over four consecutive months. In Nigeria, VAS campaigns are carried out in May/June and October/November whereas SMC administration typically happens from July to October each year. This timing shows an overlap only in October, therefore making integration possible only once a year. Thirdly, an integrated strategy will require that CDDs acquire additional skills to administer vitamin A capsules, perform additional tasks, and spend more time to complete the household visit, all of which could result in extra workload and possibly compromise the coverage of SMC or the quality of delivery. While the existing SMC programme in Nigeria provides a ready platform and an opportunity to do more with available resources, concerns remain about overloading the CDDs and their work being less effective [21, 22]. Furthermore, the feasibility and acceptability among caregivers and CDDs of integrating VAS with SMC, is unknown.

Malaria Consortium in collaboration with the National Malaria Elimination Programme (NMEP) in Nigeria, Sokoto state Malaria Elimination Agency and Sokoto state Primary Healthcare Board, carried out a study to understand the feasibility and acceptability of co-implementing VAS with SMC delivery, using one local government area (LGA) in Sokoto state. Its objectives were to assess the feasibility of integrating VAS with the SMC programme, explore the acceptability of integration from the perspectives of CDDs, caregivers, state and national-level healthcare programme officers; and estimate potential changes to the coverage and quality of SMC after integration. We conducted a pilot study of integrated VAS and SMC delivery in one LGA in Sokoto State to understand the impact, feasibility and acceptability of this strategy, and to guide future implementation and scale-up to increase VAS coverage in Nigeria.

## Methods

### Study setting and population

Sokoto state is in north-western Nigeria and SMC is currently implemented in all 23 LGAs of the state. Child health indices in the state are poor; the 2018 Nigeria Demographic and Health Survey (NDHS) indicates that only 5 percent of 6–59-month-old children received all age-appropriate vaccinations [10]. The proportion of eligible children who received at least one dose of VAS six months prior to the NNHS was estimated at 6.2 percent [12].

This study was conducted in Dange-Shuni LGA, located in the south-eastern part of Sokoto state. Based on projections from the 2006 national census report, Dange-Shuni with its eleven wards has a total estimated population of 285,697 and under-five population of approximately 57,139 [23]. SMC commenced in Dange-Shuni LGA in 2016 and coverage in 2018 was 100 percent [18]. Dange-Shuni LGA was selected because of this high administrative coverage of SMC the previous year, the existence of a pool of CDDs already trained in SMC delivery and its low risk for security challenges.

The study population was children aged 6–59 months eligible for SMC and VAS who reside in Dange-Shuni LGA. Children who fell outside this age range, those who have suffered from severe illness or allergies or had taken VAS in the month prior to the commencement of the study were excluded from this pilot implementation study.

### The pilot intervention

The pilot study leveraged on existing standard tools used for documenting VAS and SMC data separately. SMC tally sheets and referral forms were adapted to capture the required indicators for vitamin A monitoring and commodity tracking; tools for both interventions were combined. A supply-chain-management system for both drugs was set up with all the required resources and materials. CDD teams were attached to health facilities for commodity logistics and submission of reports. A designated health worker within each facility provided supportive supervision to the CDD teams attached to each health facility.

There was a training of trainers on the protocol for the integration of SMC with VAS at the state level facilitated by resource persons from the Nutrition Unit of the Federal Ministry of Health and NMEP. Subsequently CDDs and their supervisors were trained by the state trainers, using a revised training guide and job-aids. Guidelines were developed to help the CDDs determine the age of children to ensure vitamin A was administered to children aged 6–11 months and 12–59 months at the age-appropriate dose. The training focused on how to assess eligibility, how to administer VAS along with SPAQ [including the need for CDDs to wait 30 min between the administration of SMC and VAS as well as to see if the child vomited or needed SPAQ redosing], identification of adverse drug events and how to record data. Quality assurance for the training was ensured through administration of pre- and post-tests and competency assessment. Adherence to SMC dosage regimen was defined as eligible children taking the right dose of SPAQ at the right quantity and time during the SMC campaign as demonstrated by CDDs giving the first dose of SPAQ as a DOT and the caregivers giving the 2nd and 3rd day doses with proper documentation.

**Fig. 1:**
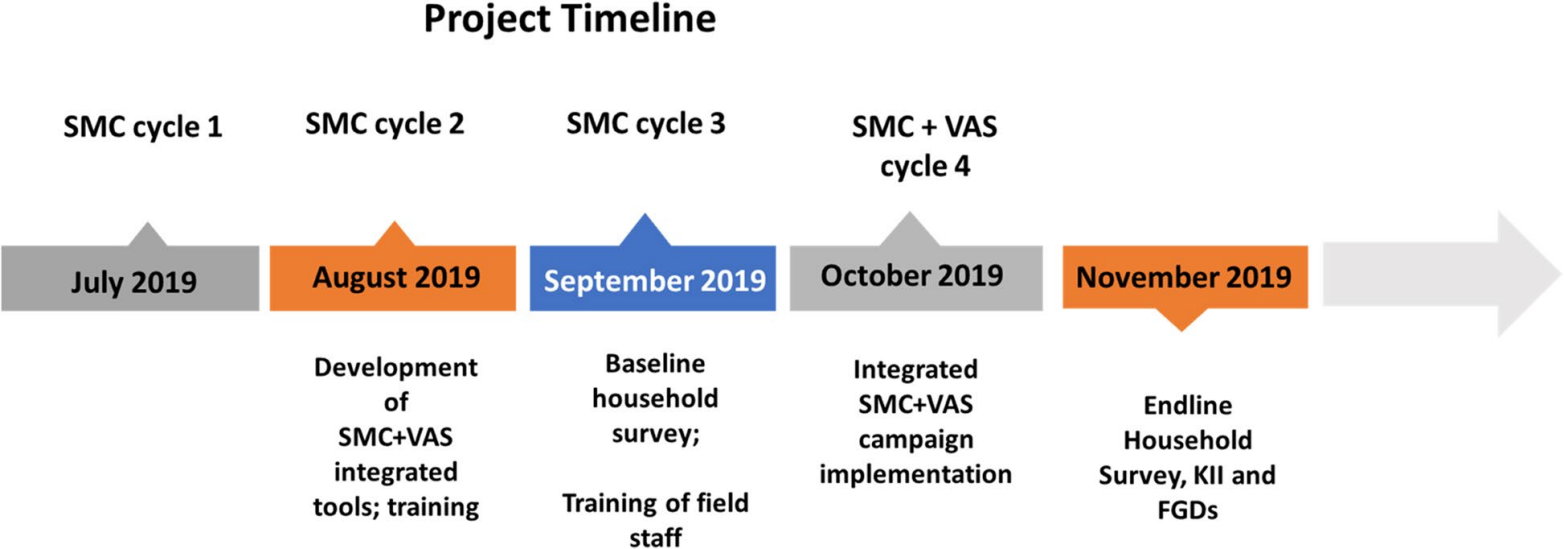
Project timelines

VAS was implemented during the last month (cycle 4) of SMC delivery i.e., October 2019. Please see Fig. 1 showing the project timelines.

The CDDs followed the same standard operating procedure for SMC with additional steps to administer vitamin A capsules to 6–59-month-old children. CDDs worked in pairs, one CDD administering SPAQ and vitamin A, while the other counted and recorded treatments in the SMC tally sheet and other tools such as the Vitamin A sticker and Child SMC card. In order to accommodate the extra workload, the daily target of the CDDs was reduced from 70 children per day to 60 children per day and more CDDs were recruited. State and LGA teams monitored activities at the health facility and in the community using standard checklists.

### Study design

This was a concurrent QUAN-QUAL mixed method study [24] In this study, collection and analysis of qualitative and quantitative data occurred at the same time and independently and both had equal status. The results were integrated at the interpretation stage, that is they reflect a combination of quantitatively established ‘impact’ of integrating VAS with SMC, and a qualitative description of the ‘feasibility’ and ‘acceptability’ of the strategy. Together the components provide different but complementary data to address the research question.

Baseline and endline surveys were used to assess the potential ‘impact’ of integrating VAS and SMC by documenting changes in coverage of Vitamin A and SMC during the study period. The qualitative component explored ‘feasibility’ and ‘acceptability’ through key informant interviews and focus group discussions with policymakers, programme officials and technical partners.

### Sampling and data collection

#### Baseline and endline surveys

To provide estimates for VAS and SMC coverage both at baseline and at endline, standardised questionnaires were administered to households with SMC-eligible children (aged 3–59 months). The survey tools were uploaded and administered on mobile android devices by trained data collectors who interviewed household heads and caregivers. Data were collected on knowledge of VAS and SMC, children receiving SMC and/or VAS and those receiving the first dose of SMC as DOT among others.

The minimum sample size of 180 children under 5 years old was obtained by using one-sample proportion formula, assuming a proportion of 6.2 percent [which represents the proportion of eligible children who received at least one dose of vitamin A six months prior to the NNHS in Sokoto state] at the 95 percent confidence level (CI) and allowing for a design effect of two. The sampling procedure involved the selection of an eligible child from each of six randomly selected households from each of three randomly selected communities within each ward. A total of 198 households were to be sampled for interview from 33 communities in all 11 wards at baseline and endline from the same catchment communities of focal health facilities in Dange-Shuni LGA.

#### Qualitative methods

To explore feasibility of the integrated VAS-SMC strategy, key informant interviews were conducted with stakeholders at national and state level (policymakers, programme managers and officials and technical partners). We used focus group discussions to explore acceptability of the strategy with CDDs and caregivers of children who received Vitamin A and SMC. Qualitative data were collected about 3 weeks after the fourth SMC cycle (18^th^ – 24^th^ November 2019).

### Key informant interviews

Key informants were identified at Federal level (relevant MoH departments and programme managers responsible for implementing malaria control and prevention), State level (representatives of state level malaria and public health programmes) and representatives of technical partner organisations (UN agencies, international donors and charities). Key informants were recruited based on their experience and understanding of the SMC programme and ability to reflect on the integrated strategy; the primary selection criterion was individuals who had participated in the planning, implementation or supervision of SMC in 2019. A list of potential interviewees was generated, and with the support of the Malaria consortium country office, individuals deemed to meet the criteria were sent an invitation and participant information sheet by email. For those who responded, the research assistants followed up to provide further information, answer questions and arranged a time for interview. Using principles for deciding saturation [25] our initial sample was at least 9 key informants (3 from each level) selected purposefully to meet our criteria (the initial sample) and we estimated a further three interviews would be needed until no new ideas emerged (the stopping criterion). After each successive interview the research team reviewed audio files and written notes to judge if data saturation had been reached, until there were three interviews without new material.

Topic guides for the key informant interviews were developed based on an existing framework for feasibility studies, and pilot tested before use [26]. Questions were broad and open-ended with prompts to explore the following topics: implementation (extent to which the integrated VAS-SMC strategy can be delivered in context, factors affecting implementation and quality of implementation); practicality (extent to which the integrated strategy can be carried out with existing resources and circumstances; integration (to what extent can VAS be integrated with the existing SMC programme); and expansion (how the SMC programme expanded to incorporate VAS and how this affected the existing SMC programme including demands on infrastructure, sustainability, disruption to programming).

### Focus group discussions

Focus group discussions were held with CDDs, their supervisors and caregivers of children who received the integrated package of SMC-VAS. We selected participants from both hard-to-reach and easy-to-reach communities within the intervention LGA. CDDs were eligible if they had been trained on SMC-VAS administration and had implemented the integrated intervention and had participated in a previous SMC cycle. Supervisors of CDDs had to be trained on SMC-VAS administration and have at least one year experience supervising CDDs to administer SMC. Caregivers were adults aged over 18, resident in the intervention LGA, with at least one child eligible to receive SMC and had received the SMC-VAS integrated intervention. Participants were selected from across the 10 LGA wards by research assistants, working closely with LGA officials, who held community meetings to provide information about the study and identify volunteers to participate. To ensure geographical spread, each FGD drew participants from clusters of 3–4 wards. We conducted separate FGDs with male and female caregivers to allow for independence in the expression of perceptions, avoiding bias by perceived gender roles. Using the same principles for deciding saturation [25], our initial sample was at least three FGDs with each stakeholder group (the initial sample) and we estimated a further FGD would be needed until no new ideas emerged (the stopping criterion). After each FGD the research team reviewed audio files and written notes to judge if data saturation had been reached.

Topic guides for FGDs were developed using a comprehensive framework for acceptability of healthcare interventions and pilot tested before use [27]. Questions were open-ended and used prompts to explore the following topics: attitude towards the intervention, burden or effort required to participate in the intervention, intervention coherence or the extent to which participants understand how it works, perceived effectiveness and confidence in participating in the intervention.

Trained research assistants conducted all FGDs and KIIs, with one acting as moderator and the other as note taker/operating the audio-recorder. FGDs took place in a quiet and neutral space in communities (usually a school building or compound), and community leaders helped to maintain privacy for the duration of the FGD. All discussions took place in local language and were audio-recoded with participant consent, with questions adapted to maximise participant understanding. Key informant interviews took place immediately after the FGDs.

### Data analysis

Baseline VAS coverage was estimated according to the proportion of eligible children who had received VAS during the MNCH week or a related intervention (such as National Immunization Plus days) in the six months preceding the study. Baseline SMC coverage refers to the proportion of eligible children who had received at least the first dose of SMC during the third cycle of the 2019 SMC campaign. Endline coverage refers to coverage achieved through the co-implementation of VAS with SMC during the fourth cycle of the SMC campaign. Frequencies and proportions were calculated for the quantitative data using STATA 15. Coverage points estimates with 95 percent CIs were calculated and compared between survey periods using cluster-adjusted chi-square tests [28].

Audio recordings of qualitative data were transcribed verbatim by the research assistants and analysed using the ‘codebook’ approach to thematic analysis with inclusive teamwork [29]. One author (HS) read a selection of transcripts and notes taken during each interview and FGD, and developed initial code lists; codes were derived inductively from the data and based on concepts in the topic guides (feasibility and acceptability). Separate code lists were developed for each participant group (CDDs, supervisors and caregivers). The initial code lists were reviewed by the research team, who suggested revisions or additional ideas. HS then used the agreed code lists to code all transcripts using MaxQDA software; inductively derived codes were incorporated as the coding progressed. Coded data were collated and exported into matrices and further interrogated to collate related codes into the main themes of feasibility and acceptability and related sub-themes, across all three participant groups.

Preliminary themes and sub-themes were reviewed by the whole team to ensure meaning and once agreed and refined themes and sub-theme were described, and comparisons made between participant groups were apparent. Table 1 shows how coded data extracts link to sub-themes and the overarching main themes.

**Table 1:** Coded data, sub-themes and themes

Main themes	Sub-themes	Coded data extracts
	Factors facilitating implementation	Key informants Less technical knowledge is needed to administer SMC and vitamin A; enough manpower to deliver the integrated programme as it has less technical requirement (compared to polio or other vaccinations) CHW structure is already in place SMC tools and forms in place Drugs generally available and logistics systems joined up Harmonise intervention timing for the integrated intervention CDDs and supervisors Given additional information about the pilot study and integration of vitamin A Training content was sufficient and taught about drug administration Community leaders important at pre-implementation stage Radio announcements about integration of vitamin A were insufficient Need to involve community leaders in pre-implementation information dissemination
	Perceived disadvantages and implementation barriers	Key informants Reliable data collection needed Need to integrate with routine health system Make sure data capture in place and harmonised The different eligibility age groups for vit A and SMC is confusing to CHWs CDDs and supervisors Some caregivers complain about ‘double medication’ Problems with data recording on tally sheets and SMC cards Caregivers Some prefer to separate administration of SMC and vitamin A
	Effect of integration on programme delivery	Key informants CHWs complain a lot about payment delays and non-payment Need to reduce SMC targets because of 30 min wait Provider workload needs adjusting Needs careful planning to avoid disruption CDDs and supervisors Longer time in the field with the 30 min wait between SMC and vitamin A Workload has increased with need to wait 30 min Explaining the integration and two drugs to caregivers takes time Concern that SMC targets not being met because of the 30 min wait No increase in payment with increased workload CHWs spend own money on transport, no allowance Payments not made, delays in payments Some won’t participate next year if payments not made Need to create separate vitamin A distribution team Caregivers Most CHWs don’t wait 30 min between administering SMC and vitamin A CHWs should be more patient with caregivers Prefer to separate administration of SMC and vitamin A
	Sustainability	Key informants State ownership important for sustainability is the implementer and should own the programme, from the beginning; state should drive the programme; needs willingness from the state for sustainability Explore use of alternative funding via corporate social responsibility /private finance Community accountability Recruit CHWs from communities Recruit more female CHWs who have better access to households CDDs and supervisors Need to select CHWs to work in their own/familiar communities; don’t accept ‘strangers’ or CHWs from out of area Caregivers Reliance on leaders to convince caregivers who reject SMC and vitamin A Community leaders should be more involved in planning and awareness raising
**B. Acceptability**	Favourable opinion of the integrated strategy	Key informants Some say it can be scaled up Integration is an innovative way of delivering, can be win–win Integration helps get vitamin A to eligible children Integration is welcomed as a vehicle for delivering more than one programme Integration is cost effective Boosts coverage and gets vitamin A to children who were missed If vitamin A is kept at facility caregivers will not come; take it to the household and they accept it Households who rejected SMC now accept it with the addition of vitamin A Demand from caregivers for bednets as well as SMC Integrate net distribution too, for better acceptance CDDs and supervisors Community realised the health benefits of both SMC and vitamin A Community accepts integration Household administration convenient Politics, religion Scepticism about the drugs Vitamin A helps acceptance of SMC Caregiver’s demand bednets as well as SMC Integrate net distribution with SMC Caregivers Generally positive initial view of integration of SMC and vitamin A Children are healthier Caregiver’s report no one refuses Only if caregiver is absent the child will miss the drugs Some caregivers previously rejected SMC Reliance on leaders to convince caregivers who reject SMC and vitamin A SMC has influenced acceptance of vitamin A
	Reports of side effects were uncommon	Key informants The community knows vitamin A and does not reject it, like the polio vaccine Households who rejected SMC now accept it with the addition of vitamin A But gatekeeprs are important to encourage those who reject the interventions, pockets of resistance exist Caregivers No complaints about side effects Sometimes children vomit after taking SMC
	Integrated SMC-VAS is an innovative way of improving access to life-saving medication	Key informants Integration is an alternative/innovative way of delivering, can be win–win Integration helps get vitamin A to eligible children Need ground-breaking innovations to change low coverage rates for vitamin A Need new ways of doing things, to address low coverage Value add/boosts coverage and gets vitamin A to children who were missed
	Demand to widen eligibility for vitamin A	Caregivers Caregivers demand vitamin A and SMC for adults/older people Demand for vitamin A and SMC in older children

### Ethical considerations

Ethical approval was obtained in September 2019 from the research ethics committee of the Sokoto state Ministry of Health (SMHOH/DHPRS/1830/VOL1) and from the Liverpool School of Tropical Medicine Research Ethics Committee (reference number: 19–075). Written informed consent was sought and obtained from each study respondent prior to commencing interviews and FGDs and study participants were assured of the confidentiality of their responses.

## Results

### Quantitative Results

During fieldwork, a total of 188 and 197 caregivers of eligible children were interviewed at baseline and endline respectively, from the 198 sampled households for the quantitative component of the study. This represents a response rate of 94.9 percent at baseline and 99.5 percent at endline.

### Key characteristics of caregivers for the selected eligible children

Almost all the caregivers of the selected children were females, married (98 percent) and mostly unemployed. Most of the caregivers who were interviewed at baseline (89 percent) and at endline (88 percent) had never attended school. Please see Table 2.

**Table 2:** Demographic characteristics of caregivers at baseline and endline

*Variable*	*Baseline* *N* = *188*	*Endline* *N* = *197*
	n (%)	N (%)
***Mean Age**** (Std. Deviation)*	29.5 (7.0)	28.0 (6.8)
***Sex***		
* Male*	3 (1.6)	2 (1.0)
* Female*	185 (98.4)	195 (99.0)
***Marital status***		
* Married*	185 (98.4)	193 (98.0)
* Single*	1 (0.5)	1 (0.5)
* Widowed*	2 (1.1)	3 (1.5)
***Occupation***		
* Farming*	1 (0.5)	3 (1.5)
* Teaching*	1 (0.5)	1 (0.5)
* Trading*	49 (26.1)	87 (44.2)
* Other*	6 (3.2)	10 (5.1)
* Unemployed*	131 (69.7)	96 (48.7)
***Ever attended school***		
* No*	167 (88.8)	174 (88.3)
* Yes*	21 (11.2)	23 (11.7)
***Relationship with selected child***		
* Mother*	181 (96.3)	193 (98.0)
* Others*	7 (1.1)	4 (1.5)

### Awareness of vitamin A and seasonal malaria chemoprevention

Caregivers’ awareness of vitamin A (those who have ever heard of vitamin A) was low at baseline (10 percent) but significantly increased at endline to 62 percent (*p* < 0.001**)** after the integration of VAS with the SMC campaign. Awareness of MNCH week was very low (4 percent) at baseline increasing to 19 percent at endline. Awareness of SMC was very high at both baseline (91 percent) and endline (97 percent) (Table 3).

**Table 3:** Caregivers’ level of awareness of vitamin A supplementation and seasonal malaria chemoprevention

***Variable***	***Baseline*** *N*= 188 (%)	***Endline*** *N* = 197 ***(%)***	χ2***(P-value)***
***Awareness of vitamin A***	10%	62%	113.16 (p < 0.001**)**
***Awareness of SMC***	91%	97%	5.57 (p = 0.061)
***Awareness of MNCH week***	4%	19%	26.78 (p = 0.021)

There was an equal distribution of male and female children at both baseline and endline and most children at baseline and endline were aged 24–59 months. (Table 4).

**Table 4:** Key characteristics of selected children at baseline and endline

***Variable***	**Baseline***N* = 188 (percent)	**Endline***N* = 197 (percent)	χ^2^**(*P*-value)**
***Sex***			
* Male*	94 (50.0)	100 (50.8)	0.02 (0.843)
* Female*	94 (50.0)	97 (49.2)	
***Age of child (months)***			
* 6–11*	19 (10.1)	13 (6.6)	1.84 (0.160)
* 12–23*	38 (20.2)	37 (18.8)	
* 24–59*	131 (69.7)	147 (74.6)	

### Coverage of vitamin A supplementation and seasonal malaria chemoprevention

Coverage of VAS at baseline was low (2 percent; 95 percent CI = 0.4—7.0) and increased at endline (59 percent; 95 percent CI = 47.0 – 70.7; *p* = 0.001) (Table 5).

**Table 5:** Comparison of Vitamin A and SMC Coverage between baseline and endline surveys

***Characteristic***	**Baseline***N* = 188	**Endline***N* = 197	χ^2^(*p*-value)
*Child received vitamin A supplement*	**n (percent)**	**n (percent)**	87.71 **(**p < 0.001)
*Yes*	3 (1.6)	117 (59.4)	
*No*	185 (98.4)	80 (40.6)	
*Child received SPAQ for SMC*	**n (percent)**	**n (percent)**	0.68 (0.4)
*Yes*	131 (69.7)	149 (75.6)	
*No*	57 (30.3)	48 (24.4)	

**Fig. 2:**
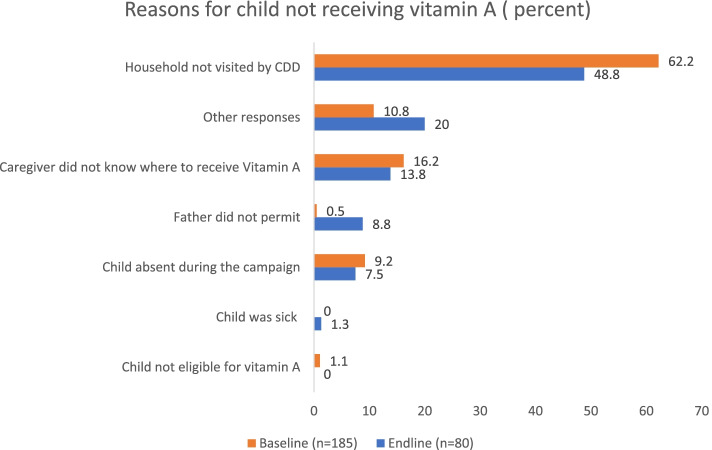
Reasons for child not receiving vitamin A (percent)

**Fig. 3:**
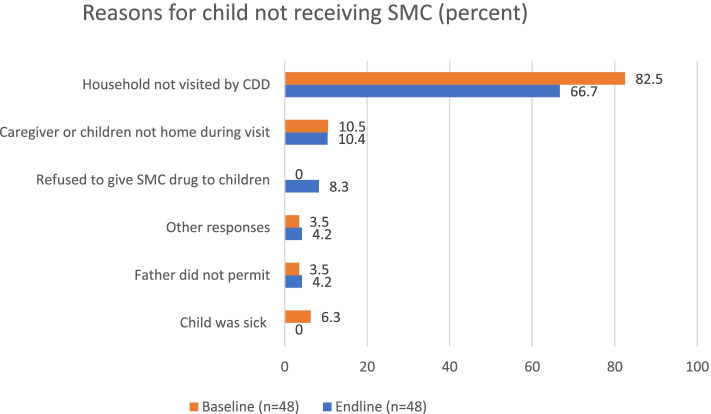
Reasons for child not receiving SMC (percent)

For children who did not receive vitamin A, the most common reason given by caregivers at both baseline and endline was that the CDD did not visit the house**.** However, this reason was given more frequently at baseline (62 percent) compared to endline (49 percent) (Fig. 2). SMC coverage increased slightly from 70 percent (95 percent CI: 57–80 percent) at baseline to 76 percent (95 percent CI: 65–84 percent) at endline (*p* = 0.4) (Table 5). Similarly, the most common reason given for not receiving SMC by care givers was that a CDD did not visit the household. Figure 3 shows that this reason was more frequent at baseline (83 percent) compared to endline (67 percent).

### Adherence to seasonal malaria chemoprevention dosage regimen at baseline and endline

Administration of the first dose of SMC by CDDs through DOT was lower at endline compared to baseline (54 percent vs 68 percent), however this difference was not statistically significant (*p* = 0.264). The administration of the second and third doses for SMC by the caregiver were similar at both baseline and endline (Table 6). Among children who received SMC, 11 out of 131 (8 percent) and 18 out of 149 (12 percent) reported having side-effects at baseline and endline respectively. The most common side-effects reported were vomiting and fever during both the baseline and endline surveys.

**Table 6:** Adherence to seasonal malaria chemoprevention dosage regimen with SPAQ at baseline and endline

*Variable*	*Baseline* *n* = *131 (%)* *95% CI*	*Endline* *n* = *149 (%)* *95% CI*	*p-value*
*Child received 1*^*st*^* dose of SP and AQ from CDD on 1*^*st*^* day *via* DOT*	86 (67.5)	80 (53.7)	0.264
*Child received 2*^*nd*^* dose of AQ at home on 2*^*nd*^* day*	126 (96.2)	149 (98.0)	0.380
*Child received 3*^*rd*^* dose of AQ at home on 3*^*rd*^* day*	126 (96.2)	142 (95.3)	0.739

### Qualitative results

Twelve key informant interviews were conducted with officials from the Federal Ministry of Health and agencies (*n *= 3), State Ministry of Health and agencies (*n *= 4), LGA Health department (*n* = 1) and donors and technical partners (*n *= 4). Twelve FGDs were conducted with caregivers (*n* = 4), CDDs (*n* = 4) and supervisors of CDDs (*n* = 4). Two overarching themes are reported, relating to feasibility and acceptability of the integrated VAS-SMC strategy. Within each theme, a series of sub-themes described participant views and observations of what is important to consider in implementing the strategy.

### Feasibility of the integrated SMC-VAS strategy

In relation to feasibility, each participant group identified factors that facilitated implementation of the integrated strategy as well as perceived implementation barriers. All groups commented on how integration affected SMC programme delivery, and key informants discussed sustainability of the VAS-SMC strategy. Each of these aspects is discussed below, using illustrative quotes.

### Factors facilitating implementation of the integrated strategy

For key informants, the most prominent factor that facilitated implementation of VAS and SMC was the fact that the SMC structure and platform was largely in place, including committed CDDs, and an established procurement process. They argued that VAS should easily be absorbed into the SMC supply chain since both ‘commodities are going to the last service delivery point’.

“*They (community drug distributors) have the passion to serve their people that is why they agreed to take the challenge*…” (State level health official, Sokoto state)

“…*we believe that the procurers of the SMC commodities should be able to support sourcing vitamin A in the long run*.” (National level key informant, Abuja)

Most national and state health officials also felt that because Vitamin A administration does not require technical knowledge, this made it easy for CDDs to undertake the work, thus facilitating its integration with SMC.

“*It does not require someone who went to school of Nursing or School of Health Technology to do the work…*” (State level health official, Sokoto state)

Community Drug Distributors (CDDs) and supervisors felt that the training they received, which they described as sufficient and included discussion about the benefits of an integrated programme, also helped with implementing the integrated strategy. They felt that the training had provided guidance on how to administer the drugs and emphasised the different age eligibility criteria for vitamin A and SMC. They mentioned being trained in ‘theory’ but also important ‘practical’ aspects such as waiting for 30 min after giving SMC before giving vitamin A and being ‘warned not to give vitamin A to children less than 6 months old’ even if the caregiver insisted.

“*When I first heard about this integration, I thought it would be difficult, administering two drugs at the same time, but with the good training, I can do it easily*” (Female community drug distributor, Dange-Shuni LGA).

Most CDDs and supervisors had heard about the addition of vitamin A to the SMC campaign through radio announcements a week before the programme started. However, there was a lot of discussion around the radio announcements being ‘insufficient’, and the need to ‘improve’ information sharing and sensitisation before the campaign. Many felt that community leaders, including imams and town announcers, should have been more involved in ‘informing and mobilising’ people to ‘cooperate’ and ‘accept’ the programme.

*“…to improve it based on the information we heard, is mobilization through community leaders, District Heads and Imams as well as town announcers for caregivers to cooperate... “(*Supervisor of community drug distributors, Dange-Shuni LGA)

### Barriers to implementation of the integrated programme

Key informants were most concerned about reliable monitoring data within the integrated strategy, especially data on vitamin A administration twice yearly. They recommended harmonised data capture, integrating new data collection tools designed for the integrated programme with existing health management information system tools, and developing one card to capture all immunizations, SMC and VAS administration.

Key informants as well as CDDs and supervisors identified that administering VAS and SMC to different eligible groups of children could cause confusion; especially since different age groups receive different dosing regimens based on their age. Caregivers also expressed concern that taking two drugs at the same time might be ‘too much’ for their children. Key informants thought it was ‘important to manage’ the different vitamin A doses for older and younger children to prevent the possibility of overdose particularly among younger children.

*“…some caregivers still feel it’s too much for a child to receive SMC and vitamin A together at the same time…” (*Female community drug distributor, Dange-Shuni LGA)

*“So, I want us to understand that deep down its useful, but we also don’t want to create confusion in the mind of the people that children who are receiving SMC for malaria are due to receive vitamin A supplement. And the dosages are different too. Children 6-11 months have different dosage compared to children from about 1 year to about 5 years, are you getting me? … you need to manage the dosages, you don’t just over dose” (National Level Key Informant, Abuja)*

Many key informants mentioned the different timing of the vitamin A administration (once a year in February and November) which falls outside of the SMC schedule (twice a year in February and November). and how this could be a barrier to integrated delivery. They suggested a need to harmonise the two interventions without disrupting ‘what is working and known’ and to avoid confusion.

### Effect of integration on programme delivery

All three participant groups expressed that workload and role of the CDDs was affected by the integration of VAS with SMC. Caregivers reported that some CDDs were not patient enough to wait 30 min, which is required between the administration of SMC and VAS to see if the child vomits; some mentioned that CDDs wait ‘just 10 min’ between SMC and vitamin A, while others ‘gave the drugs at the same time’.. Some caregivers complained that ‘some community drug distributors did not wait for children to return home’ when absent, while others ‘only asked the number of children in the household, left VAS and SMC in the household and then filled the tally cards’ – all of which goes against the guidelines provided during training.

*“…the timing is something that I know will be a problem because we know the attitude of our health workers, it is unlikely for them to wait that 30 minutes…” (*State healthcare official, Sokoto state)

CDDs and supervisors mentioned that the integrated delivery of SMC and Vitamin A was time-consuming; most CDDs were unable to meet their targets due to the additional workload, which resulted in far more time spent in the field during the campaign.

*“We wasted a lot of time. During SMC activities we close at 2 or 3pm, but with the addition of VAS we are sometimes in the field until 6pm.” (*Male community drug distributor, Dange-Shuni LGA)

“…*operationally that is what is recommended for them - to wait for like 30 minutes after giving the SMC drugs, but in practice this hardly happens, because if they are to wait 30 minutes in every house, they may end up visiting only few households…” (*National level key informant, Abuja)

A few supervisors indicated that CDDs reported being ‘stressed’ and ‘unhappy’ because the work they used to complete in 5 min (SMC) had become 30 min. Supervisors explained that the target number of children had been reduced because targets could not be met with the additional 30 min’ wait and CDDs were concerned that 4 days was now insufficient to meet their targets. To address this, caregivers and the supervisors of CDDs suggested that separate teams should be employed to administer SMC on a day different from the day the VAS is administered and key informants recommended reducing the daily targets or increasing the number of CDD teams.

By far the most discussed topic in relation to the feasibility of implementing SMC and vitamin A together were the problems CDDs had experienced with remuneration. Both CDDs and supervisors complained of ‘no increase in payment’ despite the increased workload, delays in payment and some recounted occasions when they had not been paid at all. There were strong suggestions that CDDs would not participate or ‘give their best’ in future campaigns if the payment issues were not resolved. Key informants were also concerned by complaints they had heard from the health workers and the persistent problems they faced; they found this unacceptable and suggested it would ‘demoralise’ and ‘demotivate’ CDDs.

*“When we heard about the addition of VAS to SMC, we thought payment would be increased…” (*Female community drug distributor, Dange-Shuni LGA)

*“…there are some CHWs if you asked them to come and work, they would not because they worked from cycle 1-4 without receiving any amount. If they are paid in time, the CHWs would be motivated to work. The CHWs should be paid promptly the way supervisors are paid…”* (Supervisor of community drug distributors, Dange-Shuni LGA)

*“Yes, they don’t…uhm…give them their remuneration early…yes this is one of the complaints…there is colleague that told me that he is just doing the job because he knows that is going to be beneficial to the people not because [….] of the pay…. there is a payment that has been expected, for over six months they have not paid, therefore if it’s because of the pay he will not come…”* (State level health official, Sokoto state)National and state level key informants identified that the financial and human resources required for an integrated programme would affect its delivery. They mentioned ‘logistical difficulties’ of getting materials and drugs to hard-to-reach areas and ensuring stock outs do not occur. Having adequate budget to deliver at state level was also highlighted as a major barrier to implementation.

### Sustainability of the integrated programme

Caregivers, CDDs and supervisors indicated that the sustainability of the integrated programme depended on continuous sensitization and mobilization within communities, with the help of community and religious leaders. They argued that this will increase support for the programme, help to convince caregivers who may have misconceptions about the programme, in addition to ensuring that the complete dose of VAS and SMC are administered to eligible children under five years old.

*“…creating awareness is the key to success in this programme, there is the need to mobilize and sensitize people in the community.” (*Female caregiver, Dange-Shuni LGA)

*“There is need to improve on that. Before the commencement of the program/ campaign, town announcer needs to go round the community and inform people on the upcoming activity, because some people do complain that they were not informed”* (Supervisor of community drug distributors, Dange-Shuni LGA)

*“On how to improve it based on the information we heard, it is mobilization through community leaders, District Heads and Imams as well as town announcers for the caregivers to cooperate for the acceptability of the programme”* (Supervisor of community drug distributors, Dange-Shuni LGA)

Some supervisors and key informants advised that selecting CDDs from communities in which they will be employed to distribute drugs is critical for successful implementation and community acceptability. Their rationale was that CDDs will be motivated to provide services of more value to their own communities and will be more familiar with the social and geographic landscape than those from out of area. Key informants argued that implementers should ‘employ more females than males’ as females tend to have easier access to households within communities in northern Nigeria than males due to cultural norms.

*“…on the recruitment of workers. If you consider the people of hard-to-reach areas and you pick people from such areas, they will have more confidence to do the work and the community members will cooperate*.” (Supervisor of community drug distributors, Dange-Shuni LGA)

Key informants, supervisors and the CDDs emphasized that the programme’s continuity will depend on stakeholders at different levels of the health system being involved from the start and their willingness to drive the programme. State governments should claim ownership of the integrated programme and provide the governance and accountability structures as well as the budgetary allocations required for successful implementation and sustainability. There should also be efforts to explore funding support to the government for these integrated programmes for example through public–private partnerships and corporate social responsibility initiatives from the private sector. Key informants and CDDs thought that community-level governance, stewardship and accountability were also important for sustainability and could be facilitated via the effective functioning and active participation of ward development committees (WDCs). Supervisors and CHWs thought community members should ‘cooperate’, ‘support the programme’ and ‘allow their children to receive the drugs’. They also thought community members had a role to play in ‘enlightening others’ and ‘creating awareness among themselves’. There were also suggestions about exploring alternative funding sources, including public–private partnerships and corporate social responsibility funds, instead of depending on foreign donors.

*“…sustainability has to start from government. Government must take ownership…”* (National level key informant, Abuja).

*“If it is possible, the Sokoto State Government or Local Government Areas should take the responsibility of taking the drugs to the communities. There is need for them to be involved in it…”* (Supervisor of community drug distributors, Dange-Shuni LGA)

### Acceptability of the integrated SMC-VAS strategy

All three participant groups reported a generalised ‘acceptance’ of the strategy and caregivers, CDDs and supervisors provided compelling evidence at community level. Reports of side effects or other problems with the co-administration of the two medications were uncommon, and demand from caregivers to widen the eligibility criteria for vitamin A was another indication of acceptability. Key informants praised the strategy as an innovation for improving access and coverage of vitamin A. Each of these sub-themes is described below with illustrative quotes.

### Favourable opinion of the integrated SMC-VAS programme

There was a generalised feeling among caregivers, CDDs and supervisors that communities had accepted the integrated strategy. Caregivers felt “that it is a very good and welcome development” and liked the convenience of CDDs going door-to-door within communities to give the drugs. They expressed their support for the scale-up of the integrated programme and reported that “with the intervention, the children are getting healthier”.

*“I heard nothing except appreciation about the development brought by the government to fight malaria and malnutrition among children…” (*Male caregiver, Dange-Shuni LGA)

CDDs and supervisors provided compelling examples of caregivers who were ‘very happy with the intervention’ and willing for their children to receive SMC and vitamin A. CDDs highlighted the benefits of administering VAS and SMC together and indicated that caregivers were more receptive to the integrated programme because of the perceived health benefits and the community-based door-to-door delivery approach.

“*The community members, they are very happy with the intervention, some days back I chatted with members of the community expressing their gratitude with the intervention”* (Female community drug distributor, Dange-Shuni LGA)

*“The intervention is well accepted the people were saying good thing about the programme”* (Female community drug distributor, Dange-Shuni LGA)

*“The parent and caregiver are very happy; they use to ask us what time are we coming back to administer the VAS. The intervention is useful to them they have seen the benefit”* (Female community drug distributor, Dange-Shuni LGA)Key informants also commented on the efficiency and potentially higher impact of integrating VAS with SMC.

*“…we believe that one of the major problems that we are having in primary health care is lack of integration of activities; when you have one, two, three interventions for the same age group, why not integrate it and have a better coverage than doing separate programs…”* (State health official, Sokoto state).

A common view among female caregivers was that ‘there is no child that is eligible that did not receive’ SMC and vitamin A and they had not seen anyone ‘who refused to accept it’. Children only missed or ‘skipped’ taking the drugs if the caregiver or child was absent from the household. However, it was acknowledged that some community norms for example that ‘preventive measures should not be taken’ if someone is not ill and beliefs based on rumours circulating during previous polio vaccination campaigns could lead to some community members rejecting the integrated strategy.

*“There is no child that is eligible that did not receive it”* (*Female caregiver,* Dange-Shuni LGA)

*“Yes, they do enter every house and when they do no one refuse to accept it for their children”* (*Female caregiver,* Dange-Shuni LGA)

*“There are children who were skipped because they were not around during the visit. Maybe the mother go (sic) out with the children. There is need to re-visit the households who were not around when the CDD came”* (*Female caregiver,* Dange-Shuni LGA)

Many study respondents felt that acceptability was predicated on awareness among caregivers of the health benefits of both medications. National and State healthcare officials suggested that acceptability may be due to the popularity of vitamin A and the knowledge of its health benefits among many caregivers. Caregivers too expressed that community awareness of the benefits of vitamin A had helped acceptance of the campaign.

*“…before we suffer when our children are ill and have to go to the health facility, but now the drugs are brought to the comfort of our homes…”* (Female caregiver, Dange-Shuni LGA)

*“Even when SMC was given alone it was successful and when VAS was added it improves more on the success, because before our children were always ill with malaria but since they started giving them the drugs, they became healthy”* (Female caregiver, Dange-Shuni LGA)

*“Yes, because since they started giving the drugs, we have seen our children getting healthy and better and now that VAS is added we see more improvement in their health”* (Female caregiver, Dange-Shuni LGA)

All three participant groups indicated that acceptance of the integrated programme would likely improve if mosquito nets were distributed as part of an integrated package of interventions. The absence of bednet distribution alongside SMC and vitamin A was expressed by CDDs and supervisors as a major challenge. Supervisors recommended that bednet distribution be added to the vitamin A and SMC campaign, because caregivers ‘complained’ they do not have mosquito nets yet are advised by CDDs that children should sleep under nets as well as take SMC.

*“…yes mosquito net should be added, because they keep on complaining they don’t have it , it has been shared once but did not go round, so if it can be given to them even before the program”* (Supervisor of community drug distributors, Dange-Shuni LGA)

*“The only lack of achievement is when you go into houses and they ask you for mosquito net because some of them are pregnant and they need it. So mostly that’s what they are complaining of”* (Female community drug distributor, Dange-Shuni LGA)

### Reports of side effects were uncommon

The resounding response among caregivers was there were ‘no side effects’ and that they ‘hadn’t heard anyone complain’ that their child had a problem or suffered any side effect after taking SMC and VAS. A few female caregivers mentioned that children sometimes ‘vomit’ and suffer ‘fever’, ‘weakness’ or ‘lack of energy’ after taking SMC. However, these were not seen as unusual since children typically experience such symptoms when they are vaccinated and any side-effects were quickly relieved within a day after the drugs were administered.

“*…we haven’t heard anyone that complained that their child had a problem after taking the drugs”* (Female caregiver, Dange-Shuni LGA)

*“…for some children fever is likely to occur after taking SMC while it is unlikely for other kids”* (Female caregiver, Dange-Shuni LGA)

*“…sometimes it causes lack of energy, fever and loss of appetite, but all of these symptoms happen that same day when they take the medication. By the next day, they become fine…”* (Female caregiver, Dange-Shuni LGA)

### Integration of SMC-VAS is an innovative way ofimproving access to life-saving medications

National and state healthcare officials viewed the integration as an innovation that will improve access to life-saving medications and overall, they were supportive of the programme’s scale up. Most recognised the main problem with existing health facility-based vitamin A distribution is that ‘people don’t go to the health facility’ and that house to house distribution was a ‘huge opportunity to reach out to more children and reduce the burden of vitamin A deficiency’. It was indicated that children from households in hard-to-reach communities who previously did not receive, or experienced difficulties in accessing these interventions were now covered. As a result, they felt that caregivers within the intervention communities will not have to wait for MNCH weeks or visits to health facilities to access these life-saving medications.

*“definitely…if we have not been getting the level of uptake we expect from MNCH week through VAS, why not think about different alternatives that will improve coverage…”* (National level key informant, Abuja)

*“…we thought it was very innovative, this is the first time we are considering this kind of implementation for vitamin A and malaria interventions to the same beneficiaries [under-fives]”* (National level key informant, Abuja)

### Demand to widen eligibility criteria for Vitamin A

A further indication that the integration of VAS and SMC was accepted at community level was the request from caregivers to widen the eligibility criteria. Caregivers appealed for vitamin A to be made available to children over five years old as well as adults. They argued that since vitamin A improves children’s sight, then it should be beneficial for older children and adults as well.

“*They should bring more drugs even for those children that are above 5 years...and even for adults, because we also need it”* (Female caregiver, Dange-Shuni LGA).

### “We have seen its benefit from the children who have received the drugs, please when next you want to administer you should include the adults” (Male caregiver, Dange-Shuni LGA).

## Discussion

This study was designed to assess the impact and explore the feasibility and acceptability of integrating VAS with SMC, through a pilot intervention in one LGA in Sokoto state within northern Nigeria. The increased coverage of VAS from 2 percent at baseline to 59 percent at endline suggests that an integrated SMC-VAS program provides a feasible platform to increase coverage of VAS for children under five years. This finding is similar to the result from a pilot conducted in Mali, where integration of vaccination with SMC delivery increased vaccination coverage [19]. This integration had no negative impact on SMC delivery, in line with findings from a similar SMC integration study in Nigeria [20].

Interestingly, the study also showed that SPAQ coverage marginally increased from 70 percent at baseline to 76 percent at endline following the integration of VAS with SMC. Caregivers were less likely to report that CDDs did not visit their households as a reason for children not receiving SPAQ, when VAS was integrated with SMC at endline than at baseline (67 percent vs 83 percent). Although these results were not statistically significant, exploration during FGDs and KIIs indicated that integration was viewed positively, had good reception and is acceptable at community level. These findings strengthen the argument for delivering key life-saving interventions [30] through integrated programmes, which helps to stretch available resources to deliver more dividends, especially in resource-poor setting such as sub-Saharan Africa.^13,14.15^ These findings however warrant further investigations in future studies on SMC-VAS integration.

One of the fears of integrating interventions or health service delivery is a compromise on quality of delivery following integration. This has been adduced to low adherence to quality guidelines due to limited time or extra workload [31]. However it has been established that CHWs can deliver quality services with adequate supervision, motivation and supplies [16]. The study did not show a significant difference between the percentage of children who received the first dose of SMC as DOT at baseline compared to endline following the integration (68 percent vs 54 percent). This implies the integration did not adversely affect the quality of delivery of SMC.

While some studies have shown that CHWs could take on some extra tasks without this necessarily affecting their quality-of-service delivery [32, 33], others have reiterated the direct relationship between work overload and overall productivity and effectiveness of CHWs [34, 35]. During the FGDs, some CDDs complained about the 30 min waiting time between administration of SPAQ and Vitamin A, making them spend more time in the field because of the integration despite the reduction of their daily target from 70 to 60 children per day, which could be a potential barrier in scaling up this intervention, however, this did not adversely affect their output on quality in this study. The debate about what should constitute an appropriate workload for a CDD is a long-standing one. Although there are worries on one hand about not overloading them, on the other hand, concerns remain that CHWs’ work is less effective than it could be [36] and there are ‘missed-opportunities’ [37]. CDD’s time management and efficiency while in the field is an issue worth exploring in future studies to inform scale up of the integrated campaign. Oliver et al [38] agreed that there is no question of whether CHWs can be key agents in improving health; the question lies in how to maximize their potential and do more with existing resources.

Acceptability among caregivers in this study was quite high, but acceptability of the integrated campaign could be affected by cultural norms and traditional beliefs. Lack of sensitivity to gender roles and biases when selecting CDDs could affect programme acceptability within communities, as households are likely more willing to allow female CDDs than male CDDs to enter their homes to administer the drugs because of cultural norms, especially in northern Nigeria. These should be addressed through appropriate community sensitization and mobilization as well as through targeted social and behaviour change communication messages [39, 40]. A key motivating factor for community health workers identified at organizational level by Greenspan et al., [41] amongst others, is stipend or remuneration. This study revealed dissatisfaction of the CDDs with the remuneration and the lack of promptness in paying it. It is important to determine the best remuneration packages within available resources as well as institute prompt payment mechanisms when planning for scale up to mitigate demotivation of the CDDs and ensure quality delivery of the integrated intervention.

This study also identified key success factors for an integrated SMC and VAS campaign, which include a seamless integration of data collection and field monitoring tools, adequate training of health workers and CDDs, supportive supervision, strong pharmacovigilance systems, as well as a strong coordination between key stakeholders and programme implementers at all levels during planning and delivery of the intervention. Coordination is particularly important given that the two interventions (SMC and VAS) are typically domiciled in different units of the health system—malaria programme and child health programme respectively. According to WHO, programme integration calls for a comprehensive approach with greater emphasis on prevention and the effective management of diseases as well as coordination of care using multidisciplinary teams [42].

Ultimately, sustainability and scale-up of implementation will depend on ownership by governments at different levels and their commitment to provide human as well as financial resources to support this initiative. Some key informants argued that funding support to the government could be provided through public–private partnerships and corporate social responsibility initiatives; however, this will require effective coordination and proper ring fencing of funds to be successful. Community governance structures such as the WDCs are also required to foster accountability, promote acceptability and improve ownership of the integrated programme [43].

This study is the first to investigate feasibility of integration of high dose VAS with a proven malaria intervention such as SMC. Previous VAS integration programmes were based on immunization campaigns [44, 45]. A Kenyan study reported a lower coverage for VAS following a change from an outreach-based delivery approach to a health facility-based delivery system in order to save cost [46]. The significant increase in VAS coverage demonstrated by this pilot authenticates the viability of the SMC platform to deliver VAS and potentially reach more children that are vulnerable. In 2020, over 12 million children under 5 years were reached with SMC in northern Nigeria [47], an area also known to have poor coverage of VAS [12].

### Study Limitations

It is important to bear in mind some limitations while interpreting the results of this study. Some key informants targeted were unavailable, while others involved in the study had not much knowledge of the integrated SMC-VAS programme, thereby providing limited information and feedback. Another limitation is that due to the changing security situation in the project area, the same households were not visited at endline as those sampled at baseline. Some differences in background characteristics of study participants between baseline and endline may have contributed to the differences in VAS coverage, the direction of which could not be ascertained, as there was no information about those that did not participate in the study. However, since most households in the same localities are similar, this is not likely to have affected the study in a significant way. There is also the possibility of a recall bias, especially at baseline, however responses were validated with child health cards for Vitamin A and child SMC cards for SMC where these were available.

## Conclusion

This study has demonstrated the feasibility and acceptability of co-implementing SMC with VAS. It has provided evidence that vitamin A coverage can be significantly increased when integrated with SMC campaigns without negatively affecting the quality of delivery or decreasing the coverage of SMC. The findings from this study have thus provided more evidence to support the scale up of SMC-VAS integration as a feasible and acceptable intervention to improve child survival. SMC platform could be used as a complementary platform to MNCH weeks for delivering at least one dose of VAS to eligible children annually with higher coverage. However, scaling up co-implementation will require a validation of the findings of this study on a larger scale; testing in varied contexts to tackle other potential barriers; evaluation of cost-effectiveness to inform scale-up and addressing issues identified in the study that could jeopardize future implementation.

## Data Availability

The datasets for this study are available from the corresponding author upon reasonable request.

## Abbreviations

AQ: Amodiaquine
CDD: Community drug distributor
CHW: Community health worker
CI: Confidence interval
DOT: Directly observed therapy
FGD: Focus group discussion
KII: Key informant interview
LGA: Local government area
MC: Malaria Consortium
NMEP: National Malaria Elimination Programme
SMC: Seasonal malaria chemoprevention
SP: Sulfadoxine-pyrimethamine
SPAQ: Sulfadoxine-pyrimethamine and amodiaquine
UNICEF: United Nations Children Emergency Fund
VAS: Vitamin A supplementation
WDC: Ward development committees
WHO: World Health Organisation
